# Supplementary material for: Evidence for a HURP/EB free mixed-nucleotide zone in kinetochore-microtubules
Source: Nat Commun. 2022 Aug 10;13:4704. doi: 10.1038/s41467-022-32421-x (PMC9365851; doi:10.1038/s41467-022-32421-x)

Evidence for a HURP/EB free mixed-nucleotide zone in kinetochore-microtubules

Cédric Castrogiovanni[1,2†], Alessio V. Inchingolo[3,4†], Jonathan U. Harrison[3,5], Damian Dudka[1,2,6], Onur Sen[3,4], Nigel J. Burroughs[3,5], Andrew D. McAinsh[3,4*], Patrick Meraldi[1,2*]

[1]Department of Cell Physiology and Metabolism, Faculty of Medicine, University of Geneva, 1211 Geneva 4, Switzerland
[2]Translational Research Centre in Onco-hematology, Faculty of Medicine, University of Geneva, 1211 Geneva 4, Switzerland
[3]Centre for Mechanochemical Cell Biology, University of Warwick, Coventry, UK
[4]Division of Biomedical Sciences, Warwick Medical School, University of Warwick, Coventry, UK
[5]Mathematics Institute, University of Warwick, Coventry, UK
[6]present address: Department of Biology, University of Pennsylvania, Philadelphia, USA

† Equal contribution
* Correspondence
patrick.meraldi@unige.ch - ORCID: 0000-0001-9742-8756
a.d.mcainsh@warwick.ac.uk - ORCID: 0000-0001-6808-0711

SUPPLEMENTARY FIGURES

Supplementary Fig. 1

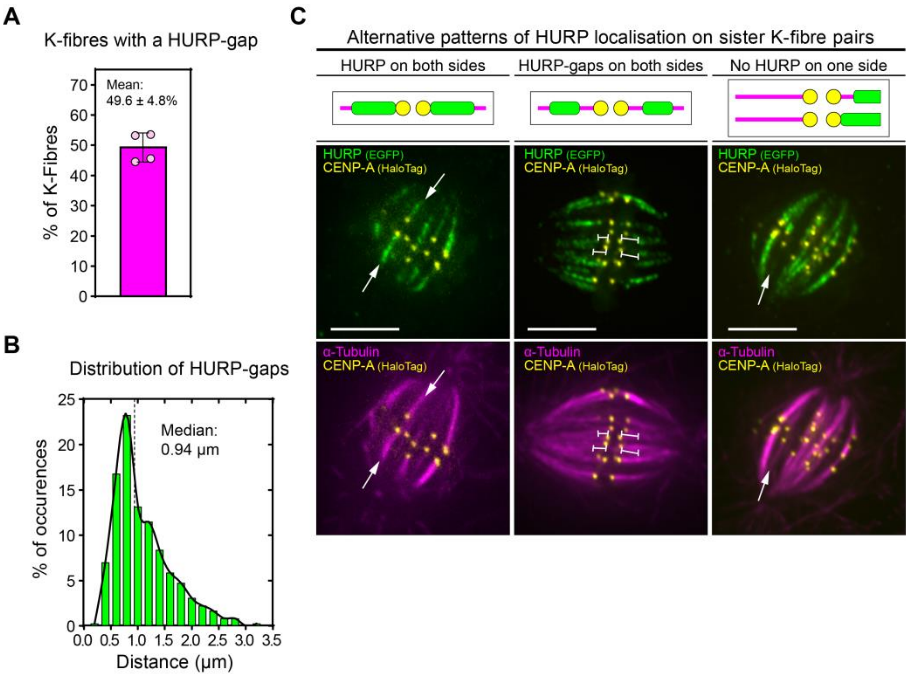

Characterization of HURP-gaps and alternative HURP localization patterns on sister K-fibre pairs in fixed cells. (**A**) Percentage of K-fibres displaying a HURP-gap in hTERT-RPE1 EGFP-HURP/HaloTag-CENP-A cells. Data are presented as mean values ± SD (N = 4, n = 838 K-fibres in 18 cells). (**B**) HURP-gap length distributions in fixed hTERT-RPE1 EGFP-HURP/HaloTag-CENP-A cells (N = 4, n = 357 gaps in 18 cells). Black line = curve fit. (**C**) Representative images of alternative HURP localization patterns along sister K-fibre pairs observed after fixation of hTERT-RPE1 EGFP-HURP/HaloTag-CENP-A cells. Z-projection of 1.9, 1.6 and 1.9 µm thickness, respectively. Scale bar = 5 µm. Source data for all graphs are provided as a Source Data file.

Supplementary Fig. 2

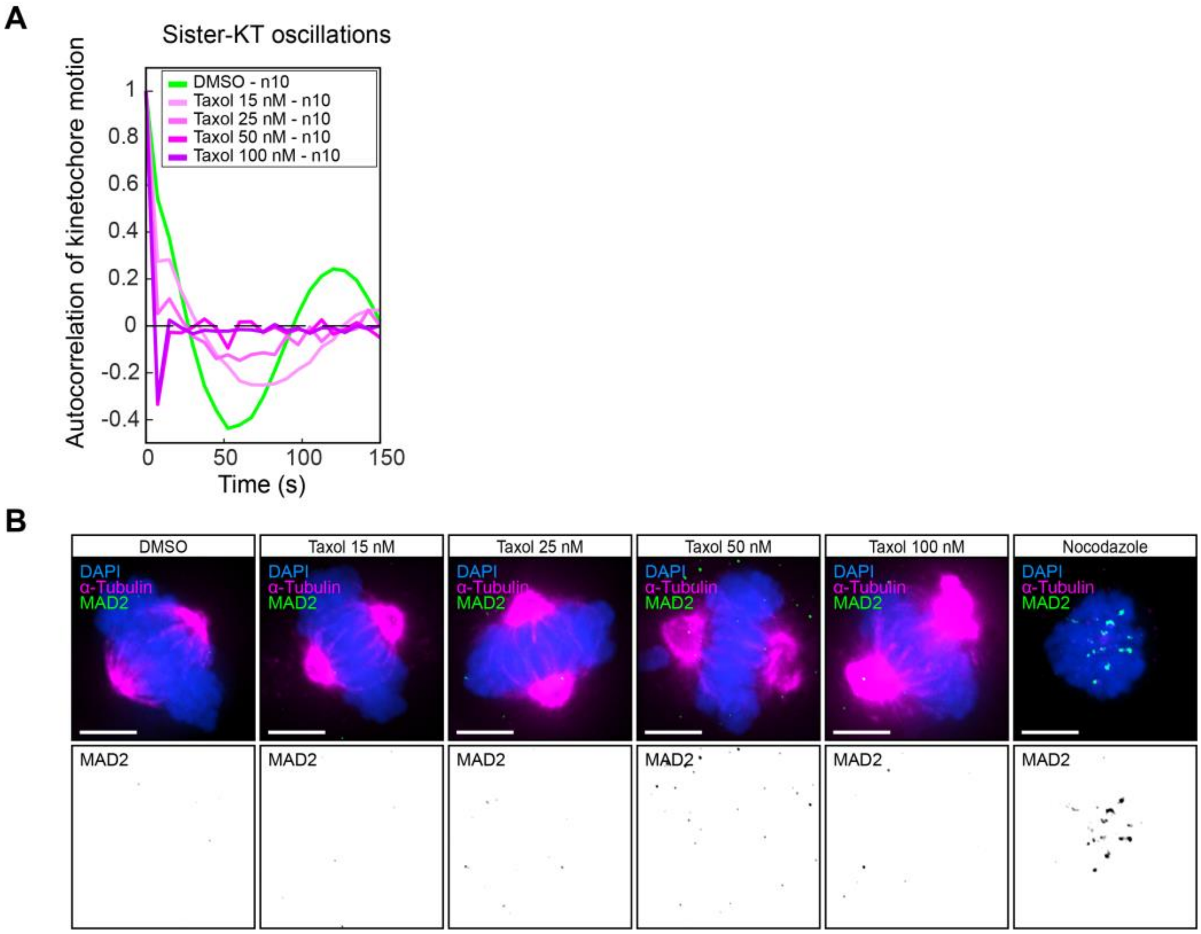

Effect of increasing taxol concentrations on kinetochore oscillations and attachment. (**A**) Autocorrelation curves of sister-kinetochore oscillations in metaphase hTERT-REP1 Centrin1-GFP/GFP-CENPA cells treated with indicated concentrations of taxol for 45 min. (**B**) Immunofluorescence images of metaphase hTERT-RPE1 cells treated with indicated concentrations of taxol for 45 min and stained Mad2. The microtubule depolymerizing drug nocodazole serves as positive control. Scale bar = 5 µm. Source data for all graphs are provided as a Source Data file.

Supplementary Fig. 3

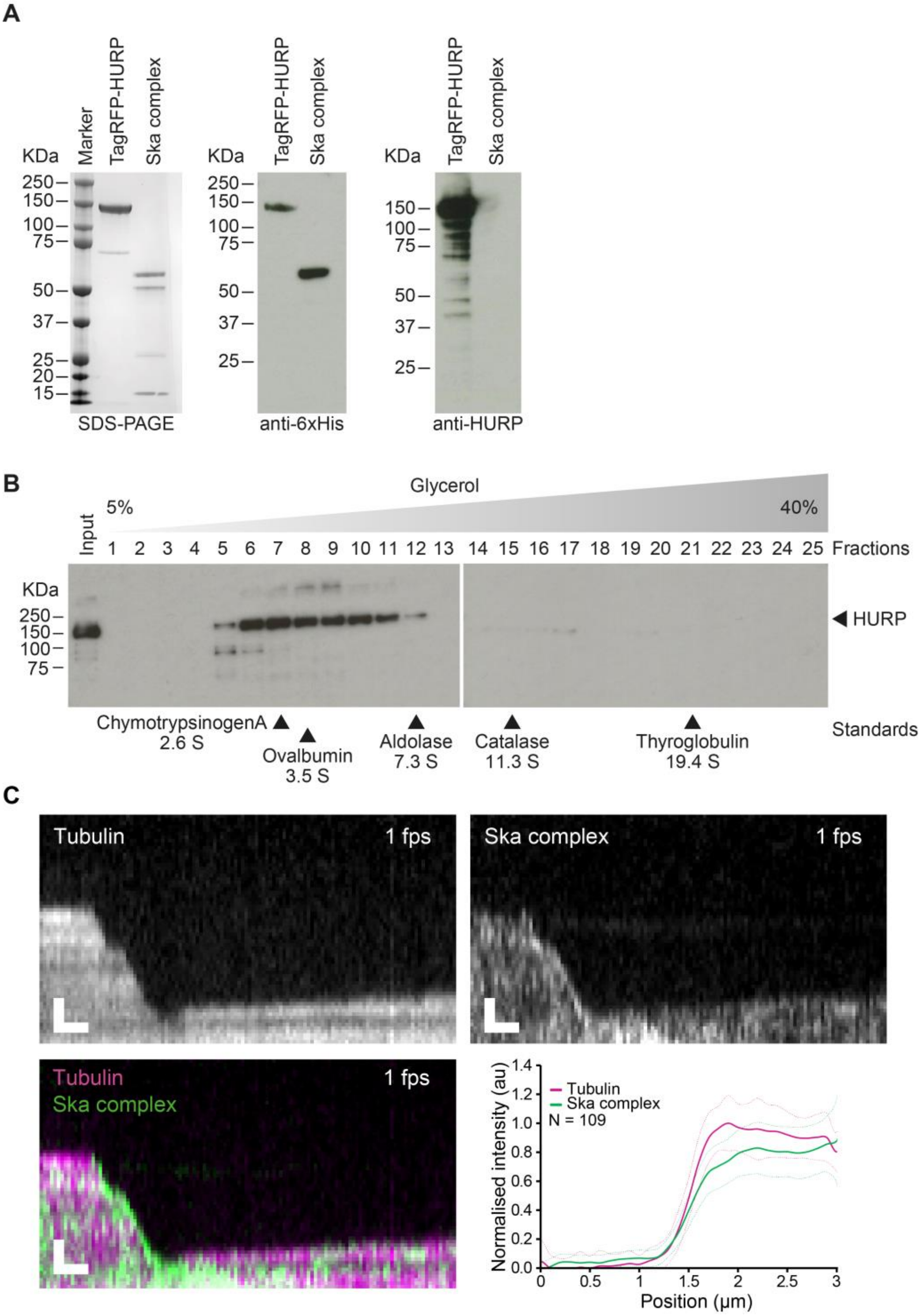

HURP and Ska complex purification (**A**) Purification of TagRFP-HURP and EGFP-Ska complex as shown by SDS-PAGE (left), anti-6xHis (middle, Qiagen penta-His) and anti-HURP (ab70744, Abcam) Western blots. The EGFP-Ska complex is used as a positive (anti-6xHis) and negative (anti-HURP) control. (**B**) Anti-HURP Western blots of 5-40% glycerol gradient fractions, indicating that the purified HURP is monodispersed. (**C**) Representative kymograph of 20 nM EGFP-Ska complex binding to and tracking a depolymerizing microtubule (left) and analysis of its mean intensity profile on the microtubules polymerizing front (N = 109); scale bars are 1 μm vertically, 10 s horizontally. Source data for all graphs are provided as a Source Data file.

Supplementary Fig. 4

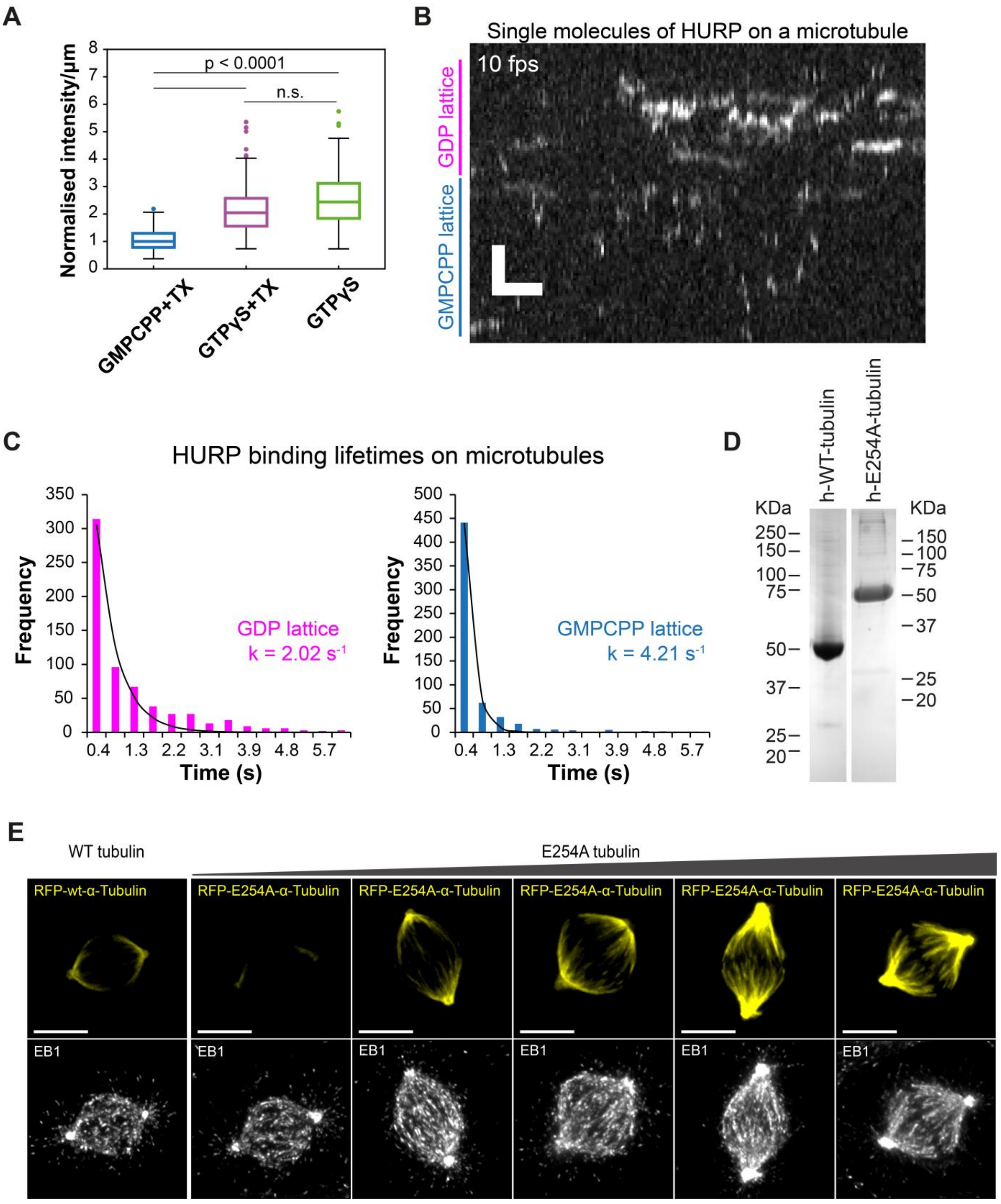

HURP preferentially binds GDP-tubulin. (**A**) Box plots of TagRFP-HURP binding to barcoded microtubules. Note that taxol did not change the amount of TagRFP-HURP bound to GTPɣS-tubulin. (boxplot indicates the 25th and 75th percentiles, the bars are medians, and the whiskers indicate values within 1.5 times the interquartile range; N = 3 flowchambers; n = 88 (GMPCPP+TX), 169 (GTPγS+TX) and 102 (GTPγS); p = Kruskal-Wallis test) (**B** and **C**) Representative kymograph of TagRFP-HURP binding on a microtubule (B) and quantification of its lifetimes (C); N = 1 flowchamber, n = 638 (GDP) and 587 (GMPCPP) HURP single molecules; scale bars = 2 µm vertically; 10 s horizontally. (**D**) SDS-PAGE gels of the purified human WT and E254A tubulin. (**E**) Immunofluorescence images of metaphase hTERT-RPE1 cells transducted either with RFP-wt-α-Tubulin or RFP-E254A-α-Tubulin and stained for EB1, arranged by amount of RFP-E254A-α-Tubulin expression level. Scale bars = 5 µm. Source data for all graphs are provided as a Source Data file.

Supplementary Fig. 5

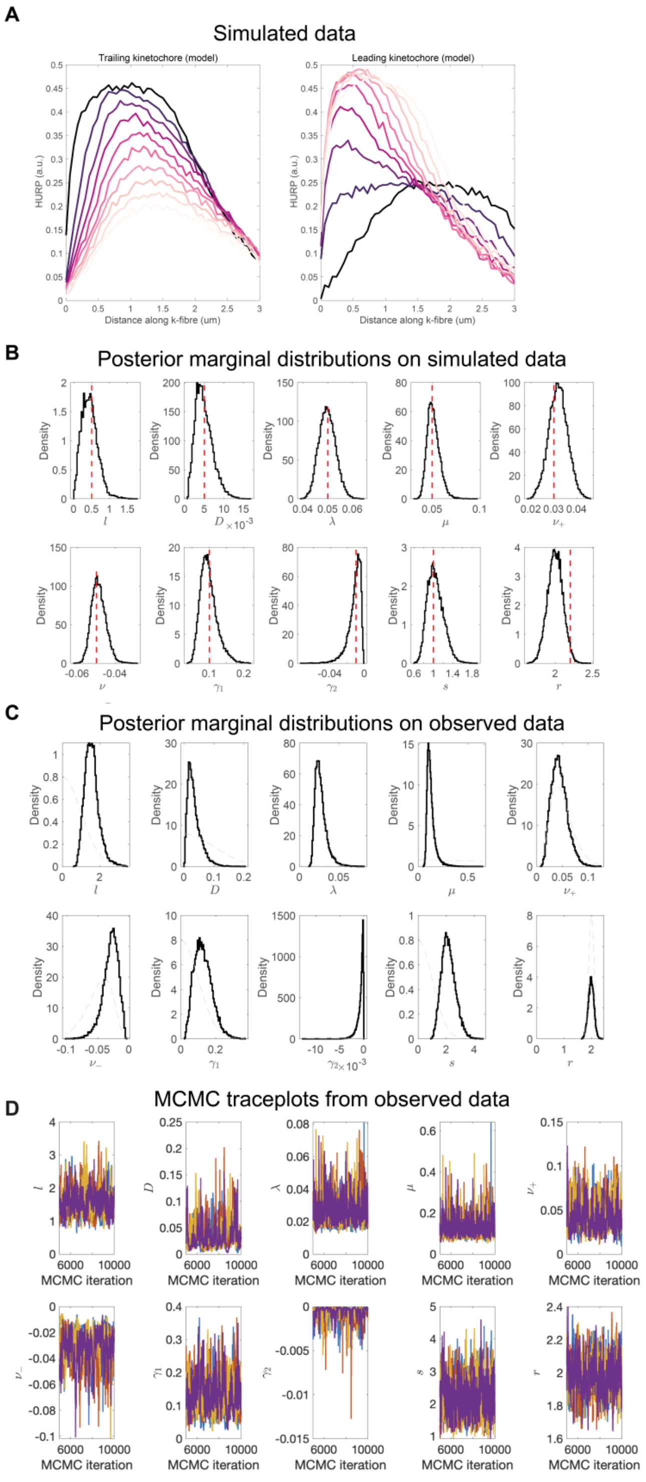

Verification of model fitting via MCMC on simulated and observed data. (A) Simulated spatio-temporal dynamics of HURP on leading and trailing K-fibres. **(B)** Posterior marginal distributions of model parameters based on the simulated data shown in (A). Red dashed vertical lines indicate the true parameters used to simulate the data. These true values lie within the mass of the posterior distribution indicating that the parameters can be identified from these data. **(C)** Posterior marginal distributions of model parameters based on observed data from lattice light sheet microscopy. As in **Fig. 6C**, but showing all inferred parameters. **(D)** Traceplots after burn-in of 4 MCMC chains (each chain shown in a different colour). Source data for all graphs are provided as a Source Data file.

Supplementary Table 1

Inferred parameters from Bayesian inference

Parameter	Median Value	95% credible interval	Prior
Size of domain (L)	3.1 μm	-	-
Final simulation time (T)	37 s	-	-
Size of GTP-cap and mixed nucleotide zone (l)	1.53 μm	[0.94, 2.46] μm	N(0,1) T[0,]
Spatial scale of RanGTP gradient (s)	2.1 μm	[1.3, 3.2] μm	N(0,1) T[0,]
HURP advection on trailing K-fibre (v_+)	42 nm/s	[19, 79] nm/s	Gamma(5,0.01)
HURP advection on leading K-fibre (v_-)	-28 nm/s	[-10, -60] nm/s	Gamma(5,0.01)
HURP diffusion constant (D)	0.029 $\mu m^2/s$	[0.006, 0.093] $\mu m^2/s$	N(0,0.1) T[0,]
HURP unbinding rate (μ)	0.12 s^{-1}	[0.08, 0.25] s^{-1}	N(0,1) T[0,]
HURP binding rate on GDP lattice (λ)	0.025 s^{-1}	[0.017, 0.046] s^{-1}	N(0,1) T[0,]
Ratio of HURP binding rate on GDP to MNZ (r)	1.99	[1.78,2.19]	N(2,0.1) T[0,]
Boundary condition at kinetochore (γ_1)	0.12 μm/s	[0.04,0.24] μm/s	N(0,0.1) T[0,]
Boundary condition at L (γ_2)	-0.0005 μm/s	[-0.0001,-0.0027] μm/s	N(0,0.1) T[0,]

Additional Supplementary File **related to Supplementary Figure 3A**
Uncropped SDS-PAGE gels and western blots

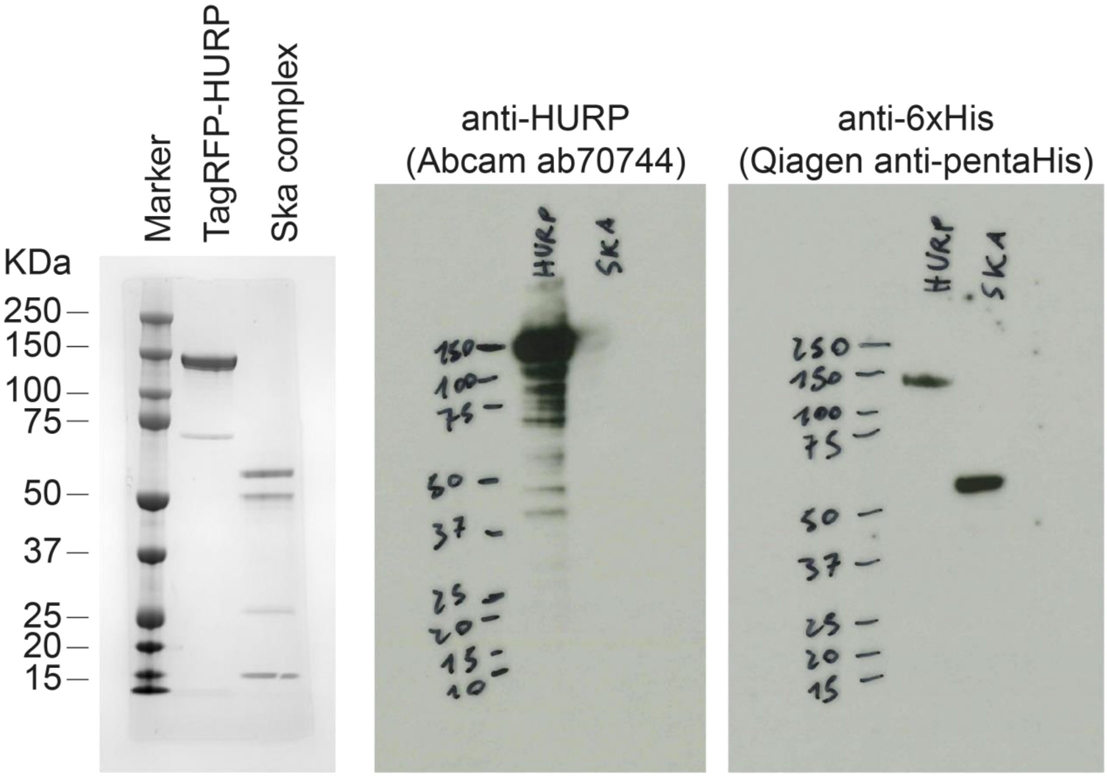

Additional Supplementary File **<u>related to Supplementary Figure 3B</u>**
Uncropped western blot

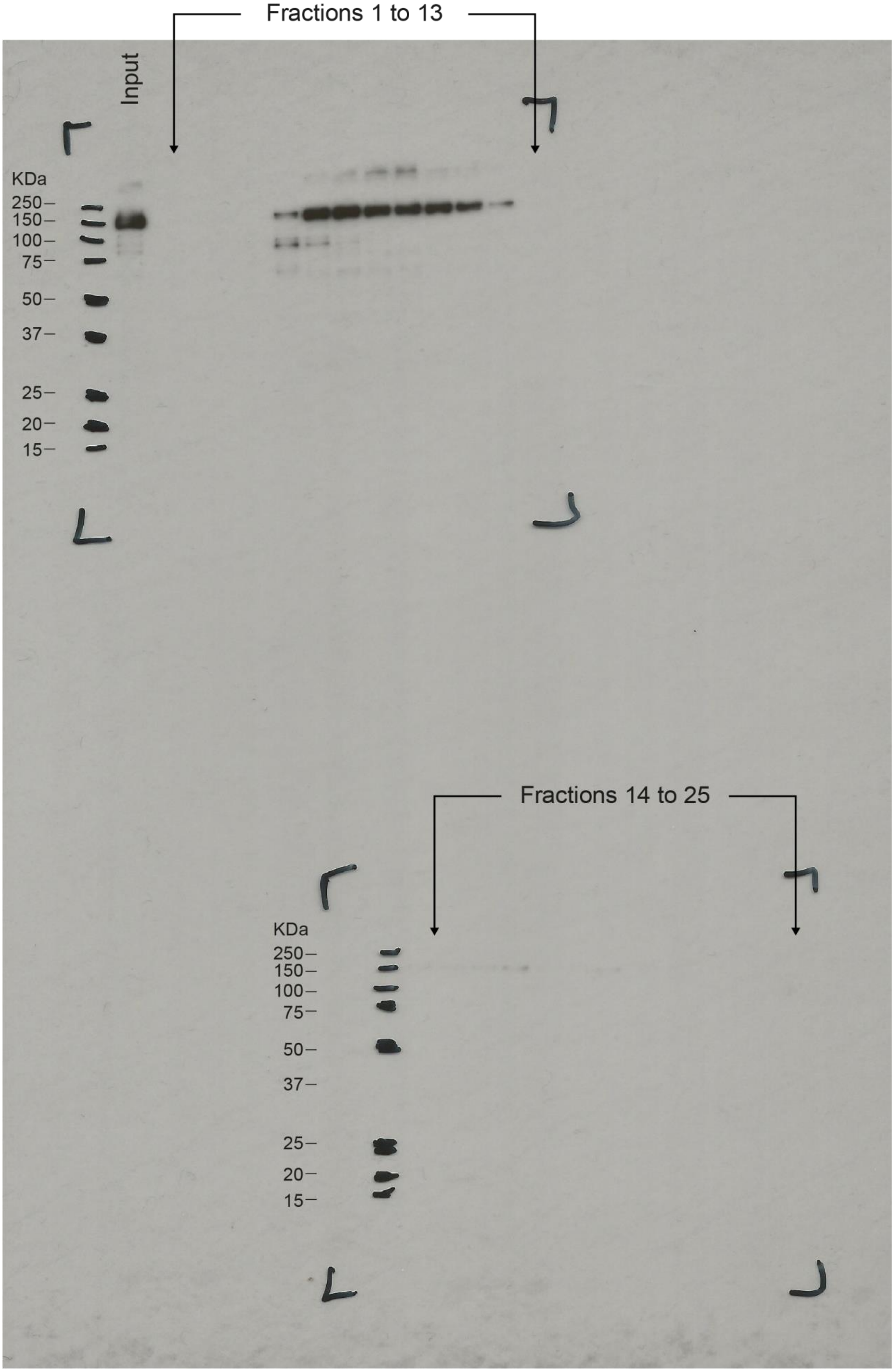

Additional Supplementary File **<u>related to Supplementary Figure 4D</u>**
Uncropped SDS-PAGE gels

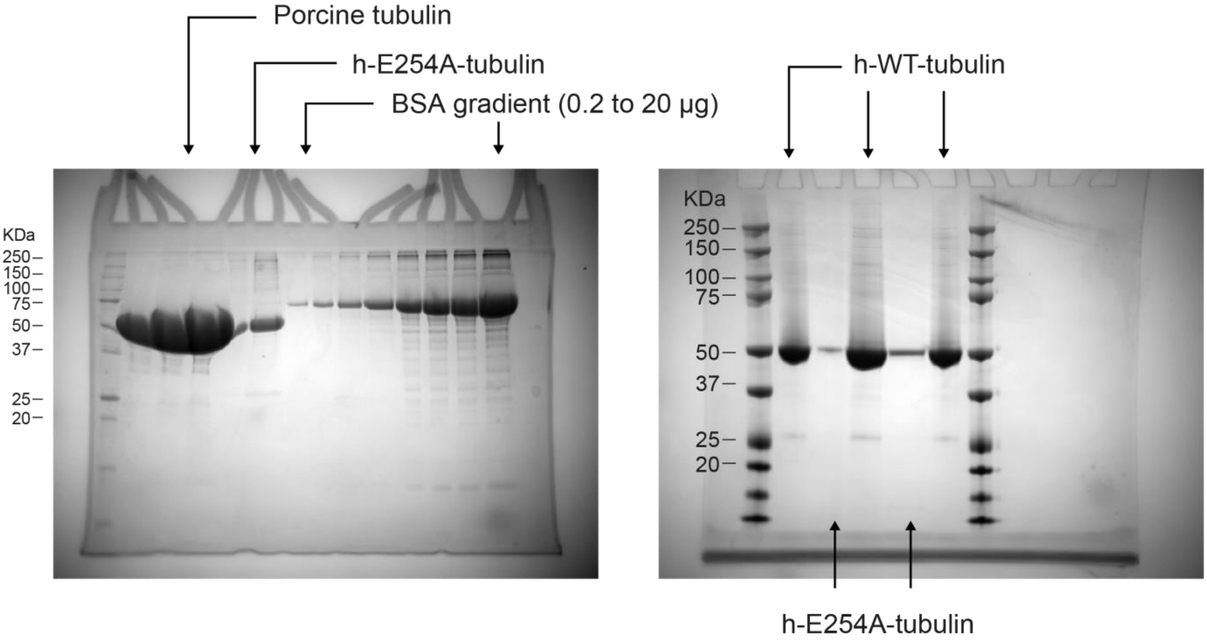

Supplement: Supplementary file 1 — Supplementary Information [file 41467_2022_32421_MOESM1_ESM.pdf]
