# Supplementary material for: Evidence for a HURP/EB free mixed-nucleotide zone in kinetochore-microtubules
Source: Nat Commun. 2022 Aug 10;13:4704. doi: 10.1038/s41467-022-32421-x (PMC9365851; doi:10.1038/s41467-022-32421-x)
Supplement: Supplementary file 3 — Description of Additional Supplementary Files [file 41467_2022_32421_MOESM3_ESM.pdf]

Description of Additional Supplementary Information

MOVIE 1. Live cell imaging of metaphase hTERT-RPE1 EGFPHURP/HaloTag-CENP-A cell.

MOVIE 2. Additional example of live cell imaging of metaphase hTERT-RPE1 EGFP-HURP/HaloTag-CENP-A cell.

MOVIE 3. Live cell image of endogenously-tagged EGFP-HURP metaphase HeLa cell overexpressing HaloTag-CENP-A.

MOVIE 4. Lattice light sheet imaging of metaphase hTERT-RPE1 EGFPHURP/HaloTag-CENP-A (Red, HURP signal; Green, CENP-A signal).

MOVIE 5. Live cell imaging of hTERTRPE1 EGFP-HURP/HaloTag-CENP-A cells blocked in metaphase (10 µM MG132) treated with DMSO or 20 µM of Parthenolide (PTL) for 90 min.

MOVIE 6. Live cell imaging of hTERTRPE1 EGFP-HURP/HaloTag-CENP-A cell line overexpressing EB3-tdTomato.

MOVIE 7. Live cell imaging of hTERTRPE1 EGFP-HURP/HaloTag-CENP-A cell line overexpressing EB3-tdTomato used to extract intensity line profiles.

MOVIE 8. Live cell imaging of hTERTRPE1 EB3-GFP cells transfected either with RFP-WT-α-Tubulin or RFP-E254Aα-Tubulin, arranged according to RFPE254A-α-Tubulin expression levels

MOVIE 9. Live cell imaging of hTERTRPE1 EGFP-HURP/HaloTag-CENP-A cells transfected either with RFP-wt-αTubulin or RFP-E254A-α-Tubulin.
